# BSD2000深部热疗联合PT方案化疗在非小细胞肺癌中的应用

**DOI:** 10.3779/j.issn.1009-3419.2010.02.10

**Published:** 2010-02-20

**Authors:** 孟祥 杨, 军 赵, 彦文 王

**Affiliations:** 252000 聊城，山东省聊城市人民医院肿瘤内科 Department of Medical Oncology, Liaocheng People's Hospital, Liaocheng 252000, China

**Keywords:** 热疗, 化疗, 肺肿瘤, 紫杉醇, 顺铂, Hyperthermia, Chemotherapy, Lung neoplasms, Paclitaxel, Cisplatin

## Abstract

**背景与目的:**

通过与PT方案单独化疗的对比观察, 探讨BSD2000深部热疗联合PT方案化疗治疗非小细胞肺癌(non-small cell lung cancer, NSCLC)的方法, 观察其近期疗效、毒副作用及生存质量改善率方面是否存在优势。

**方法:**

选择NSCLC患者60例, 随机分为治疗组和对照组, 各30例。治疗组:紫杉醇(paclitaxel, PTX)135 mg/m^2^ ivdirp 3 h qd d1+顺铂(cisplatin, DDP)20 mg/m^2^ ivdirp qd d1-5, 同时于d1、d4化疗结束后2 h内进行BSD2000热疗机精确定位热疗1 h, 21天为1周期, 共3周期。对照组:PTX 135 mg/m^2^ ivdirp 3 h qd d1+DDP 20 mg/m^2^ ivdirp qd d1-5, 21天为1周期, 共3周期, 不进行热疗。对比两组的有效率、毒副作用及生存质量改善率。

**结果:**

治疗组有效率、生存质量改善率分别为63.33%、76.67%, 对照组分别为36.67%、40.00%, 两组有效率及生存质量改善率有统计学差异(*P* < 0.05)。主要毒副作用均为骨髓抑制和消化道反应, 两组无统计学差异(*P* > 0.05)。

**结论:**

BSD2000深部热疗联合PT方案化疗治疗d可明显提高疗效, 有效率及生存质量改善率优于单纯PT方案化疗, 毒副作用可耐受。

## Abstract (en)

**Background and objective:**

The aim of this study is to determine the short-term efficacy, toxicity and the rate of life-quality improvement of BSD2000 deep hyperthermia combined with chemotherapy of PT regimen in patients with non-small cell lung cancer (NSCLC) by comparation with PT regimen alone.

**Methods:**

Sixty patients with NSCLC were randomly divided into the treatment group and control group, with 30 each.The treatment group was treated with chemotherapy (paclitaxel:135mg/m2 ivdirp 3 h qd d1+cisplatin:20 mg/m^2^ ivdirp qd d1-5) in combination with BSD2000 deep hyperthermia, and hyperthermia was positioned precisely and maintained for 60 min (2 times a cycle:d1, 4 after the end of chemotherapy within two hours).The control group was treated with chemotherapy alone.Treatment response in both groups were evaluated as well as side-effects after 3 cycles.By observing the results, comparing response rate, toxic side effects and quality of life improvement rate in two groups.

**Results:**

The efficiency and the rate of life-quality improvement in the treatment group were 63.33%, 76.67% respectively, and 36.67%, 40.00% in the control group respectively.There were significant differences between two groups (*P* < 0.05).The main side-effects were myelosuppression and gastrointestinal reactions, no significant difference between two groups (*P* > 0.05).

**Conclusion:**

BSD2000 deep hyperthermia combined with chemotherapy in patients with NSCLC can significantly increase the efficacy, response rate and quality of life improvement and without increasing sideeffects compared to chemotherapy alone.

恶性肿瘤已成为常见病、多发病, 其死亡率已跃居所有疾病死因的第二位, 其中肺癌在恶性肿瘤中的发病率及死亡率均占居首位, 而非小细胞肺癌(non-small cell lung cancer, NSCLC)又约占肺癌的80%, 化疗是治疗NSCLC的主要手段之一, 但NSCLC对化疗不甚敏感, 并且效果差、生存期短。为提高肿瘤的治疗效果, 我院引进了目前先进的美国BSD2000相控阵聚焦深部肿瘤热疗系统, 采用热疗联合化疗的方法治疗NSCLC 30例, 以期取得较好疗效。

## 1 资料与方法

### 1.1 病例选择

入组的NSCLC患者, 为无手术指征、或不愿手术治疗、或手术等治疗后复发的初次治疗者。入选条件:①经病理或细胞学检查证实为非小细胞肺癌; ②有客观观察指标, 影像学检查(CT、MRI)有可见肿瘤; ③卡氏(Karnofsky, KPS)评分为60分以上, 患者年龄≤70岁; ④近1月内未接受过其它抗肿瘤治疗; ⑤预计生存期 > 3个月, 治疗前肝肾功能检查与心电图结果均正常。将符合以上条件的患者随机分为治疗组(热疗+化疗)和对照组(单纯化疗), 每组各30例。两组病例的一般资料见表 1。

**1:** 两组病例一般资料 Patients' characteristics in two groups

Characteristics	Test group	Control group	*χ*^2^	*P*
Range	32-69	35-70	0.539^*^	0.592
Gender			0.271	0.602
Male	16	18		
Female	14	12		
KPS			0.622	0.733
60-70	8	10		
70-80	15	12		
80-90	7	8		
Primary and Recrudescence cases		0.162	0.688
Recrudescence patients	4	3		
Primary patients	26	27		
Pathological type			0.271	0.602
Sguamons carcinoma	18	16		
Adenoma carcinoma	12	14		
Stage			0.320	0.852
Ⅱ	3	4		
Ⅲ	21	19		
Ⅳ	6	7		
Test group: BSD2000 deep hyperthermia combined with chemotherapy of PT regimen; Control group: chemotherapy of PT regimen; ^*^ : *t* test.				

### 1.2 治疗方法

治疗组:化疗方案PTX 135 mg/m^2^ ivdirp 3 h qd d1+DDP 20 mg/m^2^ ivdirp qd d1-5, 同时于d1、d4化疗结束后2 h内进行BSD2000相控阵聚焦深部肿瘤热疗系统热疗1 h。21天为1周期, 共3周期。热疗采用美国产BSD2000相控阵聚焦深部肿瘤热疗系统, 其由8组偶极子天线构成环形阵列, 产生平行人体长轴线的主电场, 所有偶极子天线的辐射场通过相控阵聚集形成总的加热场。通过每组偶极子功率、振幅和相位的调配, 在人体不同深度和范围内聚焦形成理想的加热区。让患者平躺在治疗平台上, 将患者的CT或MRI数据输入计算机, 通过软件计算对治疗靶区进行定位并建立治疗计划、调节治疗平台, 使患者身体治疗靶区中心的横截面位于辐射器的中心; 将测温探针在病灶体表投射区的中心及其周围区域呈梅花状布置, 通过计算机软件进行无损测温, 给辐射器水囊充水至满, 激活治疗计划, 使初始功率为200 W-300 W, 依据测温系统数据及病人反应调整功率使病灶靶区温度保持在41 ℃-43 ℃。对照组:化疗方案PTX 135 mg/m^2^ ivdirp 3 h qd d1+DDP 20 mg/m^2^ ivdirp qd d1-5, 每21天为1周期, 共3周期, 不进行热疗。

### 1.3 评价标准

#### 1.3.1 近期疗效

两组第3周期治疗结束后及结束后4周各进行1次CT或MRI检查, 按照WHO可测量实体肿瘤的近期疗效评判标准进行疗效评判。近期疗效分为完全缓解(complete response, CR)、部分缓解(partial response, PR)、稳定(stable disease, SD)及进展(progressive disease, PD), CR+PR为有效。

#### 1.3.2 生存质量评价

用治疗前后患者一般状况KPS评分的变化反映患者生存质量的改善情况。增高:治疗后KPS评分较治疗前增加10分以上者; 下降:治疗后KPS评分较治疗前减少10分以上者; 稳定:治疗后KPS评分增加或减少不超过10分者。生存质量的评价以生存质量改善率来反映。生存质量改善率=治疗后KPS评分增高者总例数/每组总病例数×100%。

#### 1.3.3 毒性反应

按照WHO抗癌药物急性及亚急性毒性反应标准进行观察, 毒性反应分为0度-Ⅳ度。

### 1.4 统计学方法

两组间比较计数资料采用*χ*^2^检验, 计量资料采用*t*检验, 以*P* < 0.05为有统计学差异。

## 2 结果

### 2.1 近期疗效

治疗后治疗组CR 2例, PR 17例, SD 6例, PD 5例, CR+PR=19例, 有效率(response rate, RR)为63.33%;对照组CR 1例, PR 10例, SD 8例, PD 11例, CR+PR=11例, RR为36.67%。治疗组有效率明显高于对照组, 两组比较有统计学差异(*χ*^2^=4.356, *P*=0.037)。

### 2.2 毒性反应

主要为骨髓抑制、胃肠道反应, 治疗组与对照组(表 2中分别简称T和C)相比无统计学差异(*P* > 0.05)。其它不良反应为热疗时出现出汗、心跳加快和疲乏感, 但均可耐受, 且热疗结束后很快消失, 无因热疗灼伤者。

**2:** 两组患者毒性反应比较 Comparisons of toxic reactin between two groups

Toxic reaction		0	Ⅰ	Ⅱ	Ⅲ	Ⅳ	Total	*χ*^2^	*P*
Disgusting or vomitting	T	9	7	8	4	2	21 (70.00%)	0.144	0.931
	C	10	8	7	4	1	20 (66.67%)		
Diarrhea	T	25	3	0	1	1	5 (16.67%)	0.111	0.739
	C	24	4	1	1	0	6 (20.00%)		
WBC	T	16	6	4	2	2	14 (46.67%)	0.292	0.864
	C	14	7	4	2	3	16 (53.33%)		
HB	T	22	6	2	0	0	8 (26.67%)	0.089	0.766
	C	23	4	3	0	0	7 (23.33%)		
PLT	T	20	5	3	1	1	10 (33.33%)	0.364	0.834
	C	18	7	2	2	1	12 (40.00%)		
AST/ALT	T	26	4	0	0	0	4 (13.33%)	0.162	0.688
	C	27	2	1	0	0	3 (10.00%)		
Blood urea nitrogen	T	24	5	1	0	0	6 (20.00%)	0.480	0.488
	C	26	4	0	0	0	4 (13.33%)		
Serum creatinine	T	27	2	1	0	0	3 (10.00 %)	0.577	0.448
	C	25	5	0	0	0	5 (16.67%)		
Peripneral neuropathy	T	23	5	2	0	0	7 (23.33%)	0.417	0.519
	C	25	3	2	0	0	5 (16.67%)		
T: test group; C: control group.									

### 2.3 生存质量改善率

治疗后治疗组KPS评分增高者为23例, 稳定者3例, 下降者4例, 生存质量改善率为76.67%;对照组KPS评分增高者为12例, 稳定者5例, 下降者13例, 生存质量改善率为40.00%。两组相比有统计学差异(*χ*^2^=6.648, *P*=0.010), 治疗组生存质量改善率明显高于对照组。

## 3 讨论

NSCLC特别是晚期NSCLC预后差, 生存期短, 是威胁人们生命健康的主要疾病之一, 其治疗模式是综合治疗, 人们不断尝试及采用各种综合治疗模式来提高NSCLC的临床疗效, 延长患者生存时间。热疗于1985年被美国FDA认证为继手术、放疗、化疗、生物治疗之后的第五大肿瘤治疗手段^[1]^。热疗联合化疗是近年来恶性肿瘤治疗的研究热点之一, 热疗是利用物理能量在组织中积聚而产生热效应, 使肿瘤组织温度上升到有效治疗温度并维持一段时间, 以杀死癌细胞又不损伤正常细胞的一种治疗方法。同时热疗与化疗联合可增强治疗效果, 有协同作用。研究^[2]^表明热疗能改变肿瘤组织的血流灌注及增加肿瘤细胞膜的通透性, 提高化疗药在肿瘤细胞中的蓄积, 增强化疗药的细胞毒作用, 从而增强药物的抗癌效应, 体外实验加热42 ℃、2 h能使一些化疗药物抗癌效果增强10倍-100倍。Mohamed等^[3]^研究了泰索帝、紫杉醇、草酸铂、吉西他滨和美法仑在中等温度(41.5 ℃, 30 min)下对小鼠自发纤维肉瘤的细胞毒性, 发现热疗增加了泰索帝、吉西他滨的细胞毒性。

热疗还能逆转肿瘤多药耐药。冷卫东等^[4]^研究发现Tca 8113/CBDEA细胞在热疗后4 h和24 h MDR1、MRP1、GST-π耐药蛋白表达量明显下降(*P* < 0.01), Tca 8113细胞的耐药基因表达在4 h和24 h时亦有明显下降, 热疗后肿瘤细胞内阿霉素(ADM)浓度有明显上升, 表明热疗联合化疗可以逆转肿瘤细胞的耐药性, 提高治疗效果。为了解热疗联合PT方案化疗对NSCLC是否有协同治疗作用, 我们对其进行了临床研究, 结果显示热化疗组CR 2例、PR 17例, 有效率(RR)为63.33%, 对照组CR 1例、PR 10例, RR为36.67%, 治疗组有效率明显高于对照组(*P* < 0.05), 表明热疗与PT方案联合治疗NSCLC能明显提高疗效, 二者有协同治疗作用; 而且生存质量改善率热化疗组较单纯化疗组亦明显提高(*P* < 0.05), 这可能与前者疗效好有关, 同时可能也与热疗提高机体免疫力有关, 如局部热疗可使NK细胞、T淋巴细胞的活性增强等^[5]^。毒副作用二者无明显差异, 热化疗组未出现热疗常见的皮肤灼伤等副作用, 考虑与BSD-2000有先进的定位聚焦、水囊保护及测温系统有关。该研究结果表明热疗联合PT方案化疗是治疗NSCLC的有效方法, 其它临床报道也证明热疗联合化疗能提高治疗效果, 提示热疗与PT方案联合治疗NSCLC具有可行性。刘海波等^[6]^对治疗组采用盖诺+顺铂方案化疗, 每周期化疗期间联合5次局部区域热疗, 共2个周期, 而对照组则盖诺+顺铂方案单纯化疗2个周期。结果治疗组33例治疗后有效率63.6%, 对照组32例治疗后有效率37.5%。孙秀梅等^[7]^将晚期肺癌所致恶性胸腔积液的初治患者60例分为热化疗组和单纯化疗两组, 热化疗组给予吉西他滨1 000 mg/m^2^, d1、d8, 胸腔注射顺铂40 mg/次, 隔日1次, 共3次, 胸腔局部化疗24 h后患侧胸腔深部热疗, 治疗4个周期, 单纯化疗组同方案化疗4周期, 结果热化疗组控制胸水的有效率为90.0%, 单纯化疗组为66.7%。

总之, 研究表明热疗联合PT方案化疗治疗NSCLC能提高治疗效果, 可改善病人生存质量, 不增加毒副作用, 是值得推广和进一步研究的一种治疗方法。
